# Comprehensive genomic analysis of multidrug-resistant *Pseudomonas aeruginosa* isolates from companion animals in Germany

**DOI:** 10.3389/fmicb.2026.1754860

**Published:** 2026-01-28

**Authors:** Marwa Bassiouny, Hanka Brangsch, Ivonne Stamm, Peter A. Kopp, Heinrich Neubauer, Lisa D. Sprague

**Affiliations:** 1 Institute of Bacterial Infections and Zoonoses, Friedrich-Loeffler-Institut (FLI), Jena, Germany; 2 Institute of Pharmacy, Friedrich Schiller University Jena, Jena, Germany; 3 Vet Med Labor GmbH, IDEXX Laboratories, Kornwestheim, Germany

**Keywords:** antibiotic resistance, companion animals, Germany, *Pseudomonas aeruginosa*, virulence factors, whole-genome sequencing

## Abstract

*Pseudomonas* (*P*.) *aeruginosa* is a highly adaptable, opportunistic nosocomial pathogen that poses significant risks to public health and veterinary medicine. This bacterium carries a wide range of antimicrobial resistance (AMR) determinants and produces various virulence factors that enable it to invade hosts and increase disease severity. Recognised as a One Health pathogen, *P. aeruginosa* can be isolated from multiple sources, including humans, animals, food, and the environment. Despite its importance in clinical settings, there are still limited genomic and epidemiological data on *P. aeruginosa* isolates from companion animals in Germany. To address this knowledge gap, we conducted whole-genome sequencing (WGS) of 72 *P. aeruginosa* isolates collected in 2023 from various companion animals, including dogs, cats, horses, and rabbits, across Germany. Phenotypic antibiotic susceptibility testing (AST) showed that 97.2% of the isolates exhibited a multidrug-resistant (MDR) phenotype. The highest resistance rates were observed for cefotaxime (98.6%), followed by chloramphenicol (93.1%) and trimethoprim/sulfamethoxazole (87.5%). Fosfomycin resistance was observed in 26.4% of the isolates, despite all isolates carrying the *fos*A gene. All isolates were found to be susceptible to colistin at increased exposure levels. Resistance to imipenem was detected in four dog isolates. Genome analysis revealed various AMR genes associated with resistance to *β*-lactams, aminoglycosides, sulfonamides, fluoroquinolones, and phenicols. Multiple virulence-associated genes were also identified, including those involved in biofilm formation, adherence, motility, immune modulation, and exotoxin A production. Moreover, 27.8% of the isolates carried plasmid contigs, and one dog isolate harboured a class 1 integron. *In silico* multilocus sequence typing (MLST) assigned the 72 isolates to 59 distinct sequence types (STs), including five novel STs (ST5165–ST5169). The most frequently identified STs were ST253, ST258, ST395, and ST244, each represented by three isolates. Six high-risk clones (HRCs) of *P. aeruginosa*, including ST308, ST277, ST244, ST395, ST253, and ST274, were identified in dog (*n =* 8) and cat (*n =* 4) isolates. This study underscores the significant genomic diversity of *P. aeruginosa* circulating among companion animals and emphasises the need to manage this bacterium within a One Health framework.

## 1 Introduction

Antimicrobial resistance (AMR) is a growing global threat, posing significant challenges across the human, veterinary, and environmental health sectors (Naghavi et al., 2024). *Pseudomonas (P.) aeruginosa* is a member of the ESKAPE pathogens (*Enterococcus faecium*, *Staphylococcus aureus*, *Klebsiella pneumoniae*, *Acinetobacter baumannii*, *P. aeruginosa*, and *Enterobacter* species), which are recognised as major contributors to nosocomial infections and are able to escape from the biocidal effect of antimicrobials (Jadimurthy et al., 2022). The World Health Organization (WHO) has identified carbapenem-resistant *P. aeruginosa* as a high-priority pathogen that urgently needs the development of novel antimicrobials (WHO, 2024). *P. aeruginosa* is a rod-shaped, motile, Gram-negative, non-fermentative, non-spore-forming bacterium belonging to the *Pseudomonadaceae* family (Qin et al., 2022). It is ubiquitously found across diverse environmental habitats and in both human and animal hosts, where it can act as an opportunistic pathogen (Gellatly and Hancock, 2013). With one of the largest bacterial genomes (5.5–7 Mbp), *P. aeruginosa* exhibits remarkable metabolic versatility, enabling adaptation to diverse ecological niches (Klockgether et al., 2011). Its genome comprises a conserved core genome, shared by nearly all strains, and a variable accessory genome, present in some strains but absent in others (Kung et al., 2010). The accessory genome contains variable genes acquired through horizontal gene transfer, such as carbapenemases. These genes are often found on mobile genetic elements like plasmids and integrons (Botelho et al., 2019). Resistance development in this bacterium results from a combination of intrinsic and acquired mechanisms (Breidenstein et al., 2011). The intrinsic resistance of *P. aeruginosa* mainly stems from the low permeability of its outer membrane, increased efflux, and the production of antibiotic-inactivating enzymes, such as AmpC *β*-lactamase (Breidenstein et al., 2011). Acquired resistance arises from horizontal gene transfer and mutations (Breidenstein et al., 2011; Pang et al., 2019). Additionally, *P. aeruginosa’s* ability to form biofilms allows it to resist antibiotics, evade host immune responses, and survive in harsh environments (Thi et al., 2020). Furthermore, *P. aeruginosa* produces a variety of virulence factors that play crucial roles in its pathogenic process, promoting bacterial adhesion and colonisation, suppressing host immune responses, and enabling immune evasion (Liao et al., 2022). *P. aeruginosa* is a major cause of nosocomial infections, including ventilator-associated pneumonia and bloodstream infections, especially among immunocompromised individuals and patients with underlying conditions such as cystic fibrosis (Reynolds and Kollef, 2021; Tümmler, 2022). Certain *P. aeruginosa* STs, such as ST308, ST244, and ST277, are denominated as ‘high-risk’ due to their widespread presence in hospitals worldwide and their frequent association with MDR/extensively drug-resistant strains (Oliver et al., 2015).

In Germany, several nosocomial outbreaks have been linked to *P. aeruginosa* infection (Panzig et al., 1999; Pitten et al., 2001; Eckmanns et al., 2008; Schärer et al., 2023; Rath et al., 2024). In companion animals and livestock, it has been associated with various infections, including otitis externa, conjunctivitis, urinary tract infections, mastitis in dairy cows, and endometritis in horses (Haenni et al., 2015; Morris et al., 2017; Sousa et al., 2021; Nielsen et al., 2022a). An assessment by the European Food Safety Authority (EFSA) has identified *P. aeruginosa* as one of the most important AMR bacteria in the EU for dogs and cats (Nielsen et al., 2022a). The emergence of MDR and carbapenem-resistant strains, along with the co-occurrence of multiple AMR genes in companion animal isolates, has been reported in several studies (Haenni et al., 2017; Hyun et al., 2018). However, comprehensive molecular epidemiological data on *P. aeruginosa* isolates from companion animals remain limited. This study addresses this gap by using whole-genome sequencing (WGS) to characterize the genotypic, phenotypic, and virulence profiles of *P. aeruginosa* strains from companion animals in Germany.

## 2 Materials and methods

### 2.1 Bacterial isolates and identification

This study analysed 72 *P. aeruginosa* isolates obtained from IDEXX laboratories in Kornwestheim, Germany. The isolates were recovered in 2023 from companion animals across Germany. Specifically, 23 isolates (32%) originated from North Rhine-Westphalia, 10 (13.9%) from Baden-Wuerttemberg, 9 (12.5%) from Bavaria, 9 (12.5%) from Lower Saxony, 6 (8.3%) from Hesse, and 4 (5.6%) from Schleswig-Holstein. Two isolates (2.8%) each were obtained from the Rhineland-Palatinate, Brandenburg, and Thuringia. Additionally, one isolate (1.4%) originated from each of the following federal states: Saxony, Saxony-Anhalt, Berlin, Bremen, and Saarland (Supplementary Table S1). Each isolate originated from a unique sample obtained from a companion animal at a different isolation site (Table 1). Sample metadata are listed in Supplementary Table S1.

**Table 1:** Host, isolation site, number of isolates, and geographical origin of *P. aeruginosa* isolates (*n =* 72) in the current study.

Host	Isolation site	Number of isolates	Geographical origin
Dog (*n =* 58)	Ear	34	Baden-Wuerttemberg, Berlin, Bavaria, Lower Saxony, Hesse, Schleswig-Holstein, and Rhineland-Palatinate
	Wound	6	Schleswig-Holstein, Rhineland-Palatinate, Bremen, Bavaria, Hesse, and Lower Saxony
	Skin	5	Saarland, North Rhine-Westphalia, and Thuringia
	Implant	2	Saxony and Thuringia
	Vagina	2	Hesse and Baden-Wuerttemberg
	Lip	2	Schleswig-Holstein and Lower Saxony
	Nose	1	North Rhine-Westphalia
	Tail inflammation	1	Lower Saxony
	Throat	1	North Rhine-Westphalia
	Neck abscess	1	North Rhine-Westphalia
	Paw abscess	1	Saxony-Anhalt
	Tarsal region	1	Baden-Wuerttemberg
	Urine	1	North Rhine-Westphalia
Cat (*n =* 11)	Nose	4	North Rhine-Westphalia and Baden-Wuerttemberg
	Wound	2	Bavaria and Brandenburg
	Gingiva	1	North Rhine-Westphalia
	Skin	1	Baden-Wuerttemberg
	Trachea	1	Lower Saxony
	Eye	1	Baden-Wuerttemberg
	Tonsil	1	Bavaria
Horse (*n =* 2)	Trachea	1	Baden-Wuerttemberg
	Nose	1	Hesse
Rabbit (*n =* 1)	Facial abscess	1	North Rhine-Westphalia

n: number of isolates.

Microbial species identification was performed using Matrix-Assisted Laser Desorption/Ionization Time-of-Flight Mass Spectrometry (MALDI-TOF MS) with UltrafleXtreme instrument (Bruker Daltonics, Bremen, Germany) as previously described (Murugaiyan et al., 2024). Species identification was conducted using the MALDI Biotyper software according to the manufacturer’s instructions. Identification scores ranged from 0 to 3, with log scores ≥ 2.300 considered reliable for species-level identification. The genus and species identity of each isolate was confirmed using WGS data analysed with Kraken2 (v2.0.7_beta) (Wood et al., 2019), with the genus and species identified by the first match with the highest percentage.

### 2.2 Antibiotic susceptibility testing (AST)

The AST of 72 *P. aeruginosa* isolates was performed using the broth microdilution method with the automated MICRONAUT-S system (MICRONAUT, MERLIN Diagnostics GmbH, Bornheim-Hersel, Germany), as previously described by Rizk et al. (2024). Antibiotics were rehydrated with a standardized bacterial suspension adjusted to 0.5 McFarland, and 50 μL were inoculated into MICRONAUT-S MDR MRGN screening plates containing predefined antimicrobial concentrations using Mueller–Hinton broth. Plates were incubated at 37 °C for 24 h, after which minimum inhibitory concentrations (MICs) were automatically determined using a photometric reader and MICRONAUT-S software. MIC values were interpreted according to the Clinical and Laboratory Standards Institute (CLSI) guidelines (Lewis et al., 2023). The MICRONAUT-S software automatically categorised isolates as susceptible, susceptible at increased exposure, or resistant. The test panel included eight antibiotic classes, encompassing a total of 16 antibiotics, namely ciprofloxacin, levofloxacin, amikacin, colistin, chloramphenicol, fosfomycin, piperacillin/tazobactam, trimethoprim/sulfamethoxazole, piperacillin, tigecycline, ceftolozane/tazobactam, ceftazidime, ceftazidime/avibactam, cefotaxime, imipenem, and meropenem. Quality was ensured using standard reference strains *Escherichia coli* (ATCC 25922)*, P. aeruginosa* (ATCC 27853), and *Staphylococcus aureus* (ATCC 29213).

### 2.3 WGS and *in silico* detection of AMR and virulence-associated genes

Genomic DNA was extracted from pure colonies cultured overnight on Columbia blood agar at 37 °C using the High Pure PCR Template Preparation Kit (Roche Diagnostics GmbH, Mannheim, Germany) according to the manufacturer’s instructions. Sequencing libraries were prepared using the Nextera XT DNA Library Preparation Kit (Illumina, Inc., San Diego, CA, USA), and paired-end sequencing was conducted by an Illumina MiSeq sequencer (Illumina Inc., San Diego, CA, USA). The raw sequencing data were analysed using the Linux-based pipeline WGSBAC (v2.2.3, available at: https://gitlab.com/FLI_Bioinfo/WGSBAC, accessed on 15 January 2025), following the methodology outlined by Linde et al. (2020).

The raw data quality assessment was performed using FastQC, available at: http://www.bioinformatics.babraham.ac.uk/projects/fastqc/. Genome assembly was carried out using the Shovill assembler, employing the SPAdes algorithm (Bankevich et al., 2012). Species identification and potential contaminant detection were conducted using Kraken2. To identify virulence-associated genes, ABRIcate was employed in conjunction with the Virulence Factor Database (VFDB) (Chen et al., 2005). For the analysis of AMR genes, ABRIcate was used with the Comprehensive Antibiotic Resistance Database (CARD) (Jia et al., 2017), ResFinder (Zankari et al., 2012), and the AMRFinderPlus tool from NCBI (Feldgarden et al., 2020). MLST was performed using the mlst software (v2.16.1, available at: https://github.com/tseemann/mlst), applying the species-specific scheme for *P. aeruginosa* from PubMLST as established by Curran et al. (2004). Core-genome single-nucleotide polymorphisms (cgSNPs) were identified using Snippy v4.6.0,^1^ with *P. aeruginosa* PAO1 (GCF_000006765.1) or *P. aeruginosa* PA14 (GCF_025490475.1) serving as the reference genome. The phylogenetic tree was constructed using RAxML v8.2.12 (Stamatakis, 2014) based on the cgSNP alignment and visualised using Microreact (Argimón et al., 2016).

To compare the isolates with previously reported German strains, the NCBI Short Read Archive (SRA) was searched for paired-read Illumina sequencing data of *P. aeruginosa* isolates (*n =* 62) from Germany (accessed on 19 October 2024). To ensure data integrity, the datasets were examined for contamination using ConFinder (Low et al., 2019), and only uncontaminated datasets were used for further analysis. The genomic sequences obtained from the downloaded datasets were included in the cgSNP analysis alongside the isolate collection. Plasmid contigs were detected using Platon version 1.7 (Schwengers et al., 2020). All the isolates were screened for “class 1–3” integrons using *in silico* PCR^2^ with primers by Zarei-Yazdeli et al. (2018) and Delarampour et al. (2020), and the tool IntegronFinder v2.0.6 (Néron et al., 2022). Pangenome analysis was conducted using Panaroo v1.5.2 (Tonkin-Hill et al., 2020) based on genome annotations using Bakta v1.9.3 (Schwengers et al., 2021).

## 3 Results

### 3.1 Bacterial identification and whole genome sequencing data

MALDI-TOF MS identified all isolates as *P. aeruginosa* at the species level, and this identification was confirmed by WGS. Genome sequencing of all isolates yielded an average of 1,540,439 reads per isolate, with a range of 785,722 – 2,591,506. The isolates’ mean coverage was 66-fold, ranging from 34-fold to 115-fold. Genome assembly revealed genome sizes ranging from 6,204,466 bp to 7,073,023 bp. The average GC content of the sequenced genomes was 66.31%. The mean N50 value for the 72 assembled genomes was 193,539 bp, ranging from 110,797 to 380,199 bp (Supplementary Table S1).

**Figure 1:**
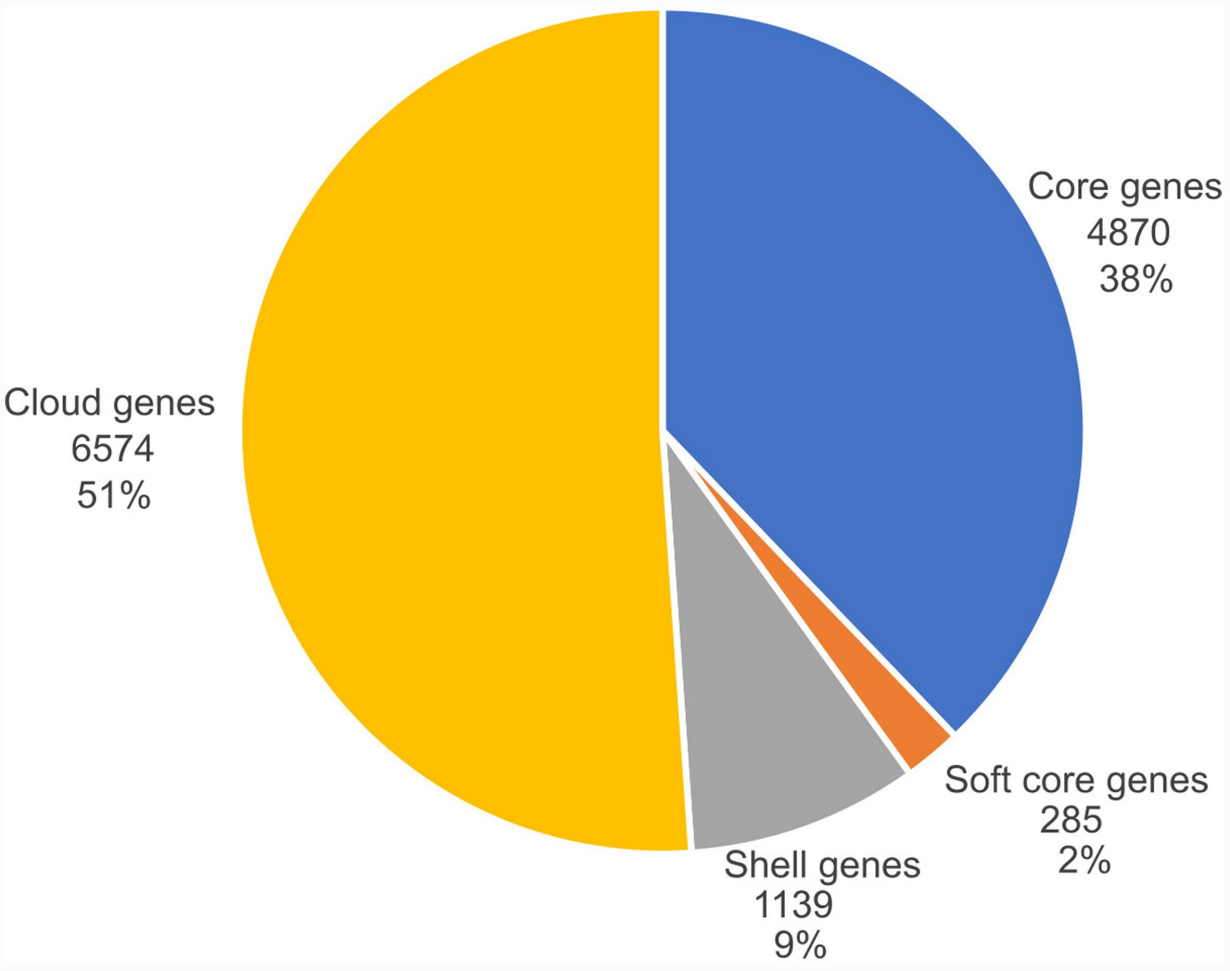
Pie chart showing the fractions of the core, soft core, shell, and cloud genes identified among *P. aeruginosa* isolates in the current study.

The pangenome analysis identified a total of 12,868 genes, distributed between a core genome (38%; 4,870 core genes) and an accessory genome (62%), which includes 285 soft core genes, 1,139 shell genes, and 6,574 cloud genes, as shown in Figure 1.

### 3.2 Antibiotic susceptibility testing (AST)

AST analysis revealed variable phenotypic resistance patterns to the tested antibiotics (Table 2). Seventy isolates (97.2%) were multidrug-resistant (MDR) (resistant to at least one antibiotic in three or more different antibiotic classes). The highest AMR rates were observed for cefotaxime (98.6%, *n =* 71), followed by chloramphenicol (93.1%, *n =* 67), trimethoprim/sulfamethoxazole (87.5%, *n =* 63), and fosfomycin (26.4%, *n =* 19). Four dog isolates (5.6%) were imipenem-resistant. All isolates were susceptible to piperacillin, piperacillin/tazobactam, ceftazidime, ceftazidime/avibactam, and ceftolozane/tazobactam. All isolates were phenotypically susceptible at increased exposure to colistin.

**Table 2:** Antibiotic susceptibility profiles of the *P. aeruginosa* (*n =* 72) isolates in the current study.

Antibiotics	Concentration, μg/mL	R (%)	I (%)	S (%)
Ciprofloxacin	0,25–2	6 (8.3%)	3 (4.2%)	63 (87.5%)
Levofloxacin	0,5–2	8 (11.1%)	6 (8.3%)	58 (80.6%)
Amikacin	4–32	0	1 (1.4%)	71 (98.6%)
Colistin	1–8	0	72 (100%)	0
Chloramphenicol	8–18	67 (93.1%)	5 (6.9%)	0
Fosfomycin	32–128	19 (26.4%)	23 (31.9%)	30 (41.7%)
Tigecycline	0,25–4	71 (98.6%)	0	1 (1.4%)
Trimethoprim/sulfamethoxazole	1/19–4/76	63 (87.5%)	0	9 (12.5%)
Piperacillin	8–16	0	0	72 (100%)
Piperacillin/tazobactam	4/4–64/4	0	0	72 (100%)
Cefotaxime	1/4–8/4	71 (98.6%)	1 (1.4%)	0
Ceftazidime	1–128	0	0	72 (100%)
Ceftazidime/avibactam	1/4–16/4	0	0	72 (100%)
Ceftolozane/tazobactam	1/4–8/4	0	0	72 (100%)
Imipenem	1–8	4 (5.6%)	2 (2.8%)	66 (91.7%)
Meropenem	0,125–128	0	1 (1.4%)	71 (98.6%)

R: resistant, I: susceptible at increased exposure, S: susceptible. Note: values represent the number of isolates in each category.

### 3.3 *In silico* detection of MLST, AMR, and virulence genes

**Figure 2:**
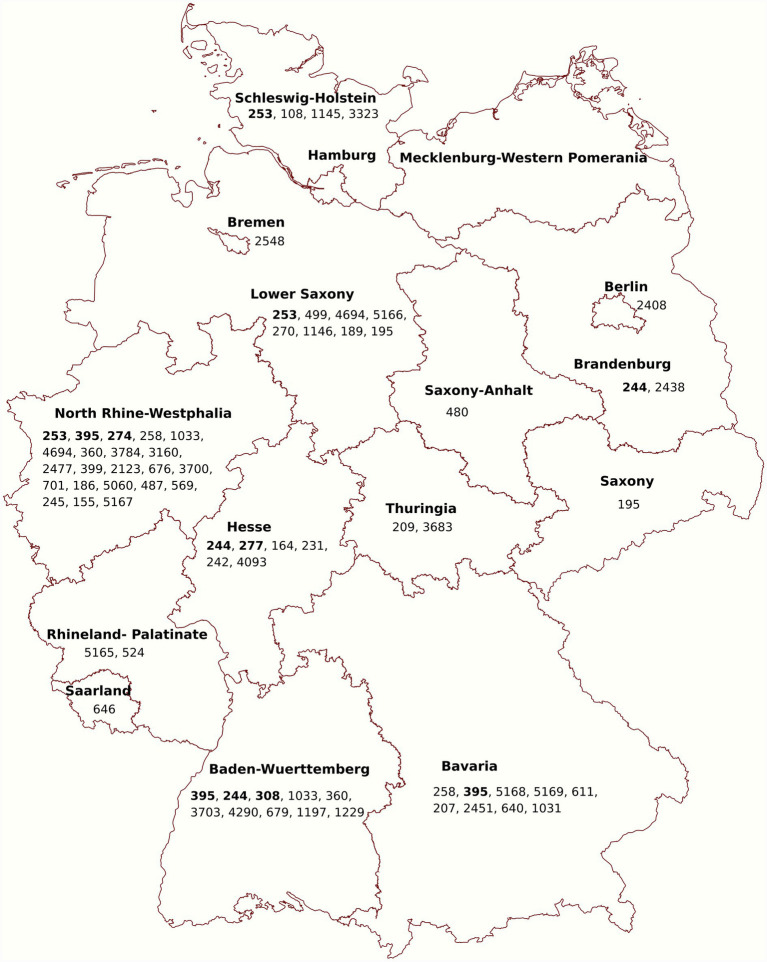
Geographical distribution of *P. aeruginosa* STs, including high-risk clones (shown in bold), identified in companion animal isolates across Germany in this study. Numbers on the map represent the STs detected in each federal state. Figure has been created by www.mapchart.net and Inkscape.

MLST analysis assigned the isolates to 59 sequence types (STs); of them, five were novel STs (ST5165-ST51699; PubMLST database, accessed on 15 November 2024). The novel STs were isolated from wound and ear swabs of dogs across Rhineland-Palatinate, Lower Saxony, North Rhine-Westphalia, and Bavaria. The predominant STs among all isolates were ST253, ST258, ST395, and ST244, each found in three isolates. These were followed by ST195, ST1033, ST499, ST4694, and ST360, each identified in two isolates. Each of the remaining STs was represented by a single isolate (Supplementary Table S1). MLST analysis furthermore revealed that 12 MDR *P. aeruginosa* isolates could be assigned to 6 high-risk clones (HRCs) of *P. aeruginosa* STs. These included ST308 (*n =* 1), ST244 (*n =* 3), ST277 (*n =* 1), ST395 (*n =* 3), ST253 (*n =* 3), and ST274 (*n =* 1). These STs were identified in dog (*n =* 8) and cat (*n =* 4) isolates as shown in Table 3. The geographical distribution of the identified STs, including HRCs, in the current study is shown in Figure 2.

**Table 3:** High-risk clones of *P. aeruginosa* identified in the current study.

MLST	Isolate ID	Host	Sampling site	Geographical origin
ST308	23Y0170	Cat	Nose	Baden-Wuerttemberg
ST244	23Y0066	Dog	Ear	Brandenburg
	23Y0115	Dog	Vagina	Baden-Wuerttemberg
	23Y0147	Dog	Ear	Hesse
ST277	23Y0162	Dog	Vagina	Hesse
ST395	23Y0169	Cat	Skin	Baden-Wuerttemberg
	23Y0171	Cat	Nose	North Rhine-Westphalia
	23Y0174	Cat	Wound	Bavaria
ST253	23Y0148	Dog	Ear	Lower Saxony
	23Y0150	Dog	Ear	North Rhine-Westphalia
	23Y0154	Dog	Ear	Schleswig-Holstein
ST274	23Y0063	Dog	Nose	North Rhine-Westphalia

MLST, multilocus sequence typing, ST: sequence type.

Genome analysis identified 80 AMR genes associated with various resistance mechanisms, including antibiotic inactivation, efflux pumps, antibiotic target alteration, and decreased antibiotic permeability. These genes confer resistance to several antibiotic classes, including *β*-lactams, aminoglycosides, fluoroquinolones, fosfomycin, and sulfonamides. The number of resistance genes per isolate ranged from 46 to 51, with the highest prevalence observed in a cat isolate from Bavaria (ST640). Thirty-nine efflux pump-associated genes were identified, and 36 of them were present in all isolates. All isolates harboured the *cat*B7 gene conferring resistance to phenicols, the *fos*A gene associated with fosfomycin resistance, the *arn*A and *bas*S genes, conferring resistance to peptides, and the *bcr*-1 gene associated with bicyclomycin resistance. A total of 32 β-lactamase resistance genes were identified. The most prevalent was *bla*_PDC.374_, identified in 65% (47/72) of the isolates, followed by *bla*_PAO-1_, found in 40.3% (29/72) of isolates. The *bla*_PDC.19a_ gene was detected once in an HRC cat isolate (ST308), while the *aad*A7 gene, linked to aminoglycoside resistance, and *sul*1 associated with sulfonamide resistance, were identified exclusively in a HRC dog isolate (ST253). The fluoroquinolone resistance gene *crp*P was found in 34 isolates. Five aminoglycoside resistance genes were identified: *aph*(3′)-IIb, *aph*(3″)-Ib, *aph*(3′)-Ia, *aph*(6)-Id, and *aad*A7. The *aph*(3′)-IIb gene was present in all isolates, while *aph*(3″)-Ib, *aph*(3′)-Ia, and *aph*(6)-Id, were detected only in a cat isolate from Bavaria. Although the *fos*A gene was detected in all isolates, only 30 exhibited phenotypic resistance to fosfomycin. Seven isolates harboured a point mutation (V15I) in the sensor kinase gene pmrB, a component of the pmrAB two-component regulatory system, which can influence colistin susceptibility. However, all isolates remained phenotypically susceptible at increased exposure to colistin (Supplementary Table S1). Further, genome analysis identified 321 virulence-associated genes. The genes associated with effector delivery systems were the most prevalent (*n =* 111), followed by nutritional/metabolic factors (*n =* 54), motility (*n =* 53), adherence (*n =* 43), biofilm formation (*n =* 33), immune modulation (*n =* 24), exotoxin (*n =* 2), and exoenzyme (*n =* 1). The number of virulence-associated genes per isolate ranged from 249 to 313, with the highest count (*n =* 313) observed in an HRC (ST244) isolate from a dog in Hesse. The Type III secretion system (T3SS) effector genes were identified, including *exo*U, detected in eight isolates, four of which belonged to *P. aeruginosa* HRCs (ST253 and ST308). The pore-forming toxin gene *exl*A was detected in four isolates, whereas the *tox*A gene was present in 63 isolates. Detailed information is provided in Supplementary Table S1.

### 3.4 Detection of plasmids and integrons

WGS using Platon predicted that 27.8% (20/72; 18 from dogs and 2 from cats) of the isolates harboured a total of 24 plasmid contigs, with the number of contigs varying among isolates (Table 4). The plasmids ranged in size from ~3.3 to 174 kbp. Isolates carrying plasmid contigs belonged to various STs, with 25% (5/20) identified as HRCs (ST277, ST274, ST253, ST244). Further AST analysis showed that 19 of 20 of these isolates demonstrated an MDR phenotype. In addition, all 20 isolates carried 47–50 AMR genes and 249–312 virulence genes per isolate.

**Table 4:** Plasmid contigs distribution among *P. aeruginosa* (*n =* 72) isolates in the current study.

Isolate ID	Host	Geographical origin	Number of plasmid contigs	Total Size of the assembly (kbp)	Size of plasmid (kbp)	MLST
23Y0167	Cat	Baden-Wuerttemberg	1	6821.715	83.5	ST1229
23Y0143	Dog	Lower Saxony	1	6622.565	49.3	ST5166
23Y0162	Dog	Hesse	1	7073.023	161.4	ST277
23Y0149	Dog	Bavaria	1	6371.522	3.3	ST5168
23Y0139	Dog	North Rhine-Westphalia	2	6305.691	19.9; 14.3	ST676
23Y0063	Dog	North Rhine-Westphalia	1	6582.126	5.5	ST274
23Y0154	Dog	Schleswig-Holstein	3	6964.613	174; 49; 5	ST253
23Y0065	Dog	Baden-Wuerttemberg	1	6522.059	6	ST4290
23Y0081	Dog	Lower Saxony	1	6413.737	5.5	ST4694
23Y0144	Dog	Hesse	1	6477.945	15.7	ST231
23Y0151	Dog	Bavaria	1	6494.728	35.2	ST5169
23Y0141	Dog	North Rhine-Westphalia	1	6570.609	31.3	ST3700
23Y0155	Dog	North Rhine-Westphalia	1	6361.100	5.5	ST5060
23Y0113	Dog	Thuringia	2	6502.645	51.5; 48.7	ST3683
23Y0075	Dog	North Rhine-Westphalia	1	6413.548	108.1	ST2477
23Y0168	Cat	North Rhine-Westphalia	1	6794.895	38.9	ST245
23Y0157	Dog	North Rhine-Westphalia	1	6568.791	6.7	ST487
23Y0148	Dog	Lower Saxony	1	7014.689	42	ST253
23Y0115	Dog	Baden-Wuerttemberg	1	6830.728	59.7	ST244
23Y0138	Dog	Saarland	1	6434.652	31	ST646

kbp, kilobase pair; MLST, multilocus sequence typing; ST, sequence type.

*In silico* PCR analysis identified one isolate from a dog in Lower Saxony that harboured a class 1 integron carrying *sul*1 (associated with sulfonamide resistance) and *aad*A7 (associated with aminoglycoside resistance) genes. This isolate also carried plasmid contigs and belonged to the *P.aeruginosa* HRC ST253.

### 3.5 Phylogenetic analysis of *P. aeruginosa* isolates

**Figure 3:**
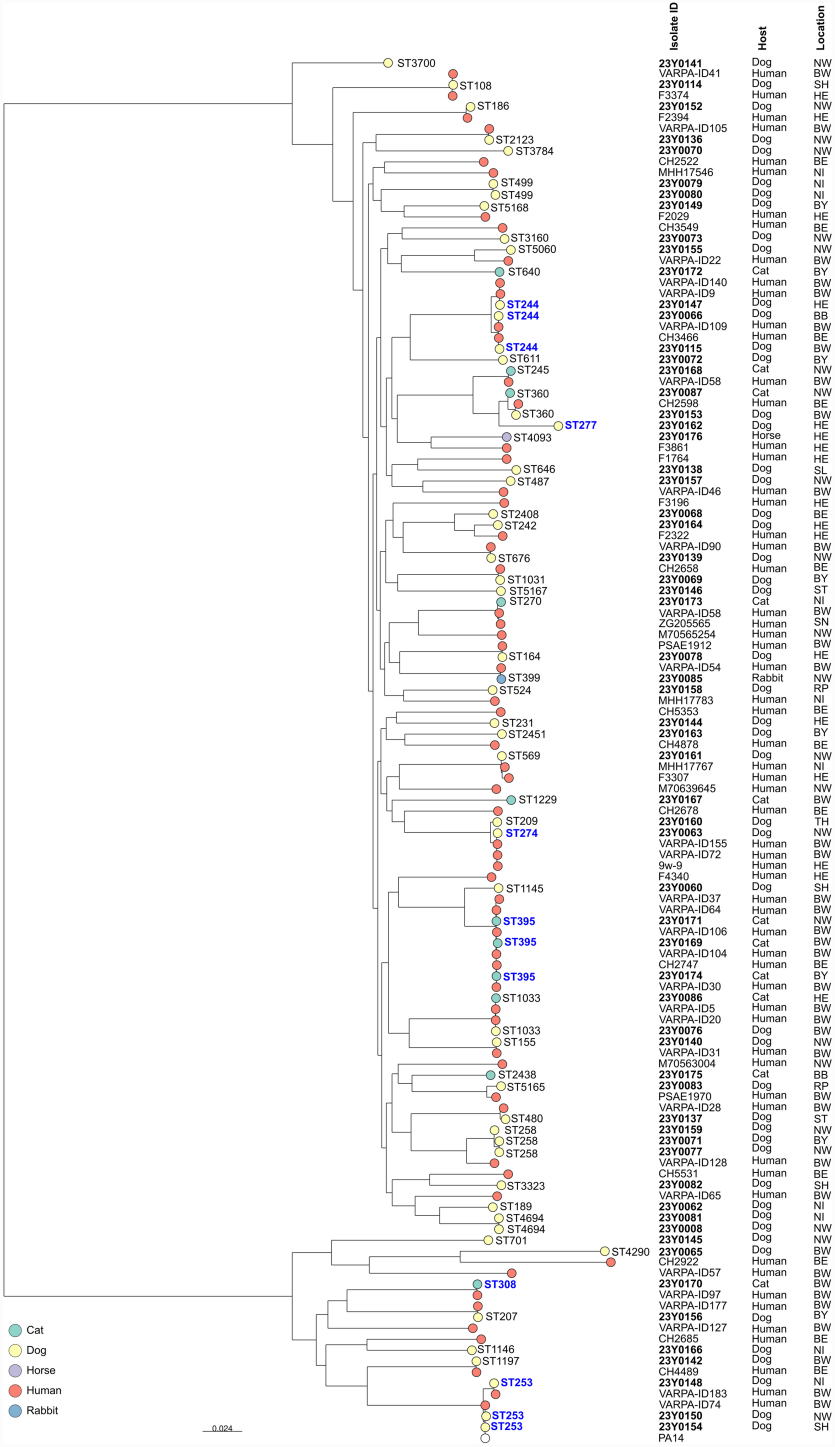
Maximum likelihood phylogenetic tree of *P. aeruginosa* isolates from Germany based on cgSNP. Leaf colors indicate the host. Isolates from this study are shown in bold, MLST sequence types (STs: 3700, 108, 186, 2,123, 3,784, 499, 5,168, 3,160, 5,060, 640, 244, 611, 245, 360, 277, 4,093, 646, 487, 2,408, 242, 676, 1,031, 5,167, 270, 164, 399, 524, 231, 2,451, 569, 1,229, 209, 274, 1,145, 395, 1,033, 155, 2,438, 5,165, 480, 258, 3,323, 189, 4,694, 701, 4,290, 308, 207, 1,146, 1,197, and 253) are indicated and HRCs are highlighted in bold blue. The bar at the bottom indicates base changes per site (BW: Baden-Wuerttemberg, BY: Bavaria, BE: Berlin, BB: Brandenburg, HE: Hesse, NI: Lower Saxony, NW: North Rhine-Westphalia, RP: Rhineland-Palatinate, SL: Saarland, SN: Saxony, ST: Saxony-Anhalt, SH: Schleswig-Holstein, TH: Thuringia).

**Figure 4:**
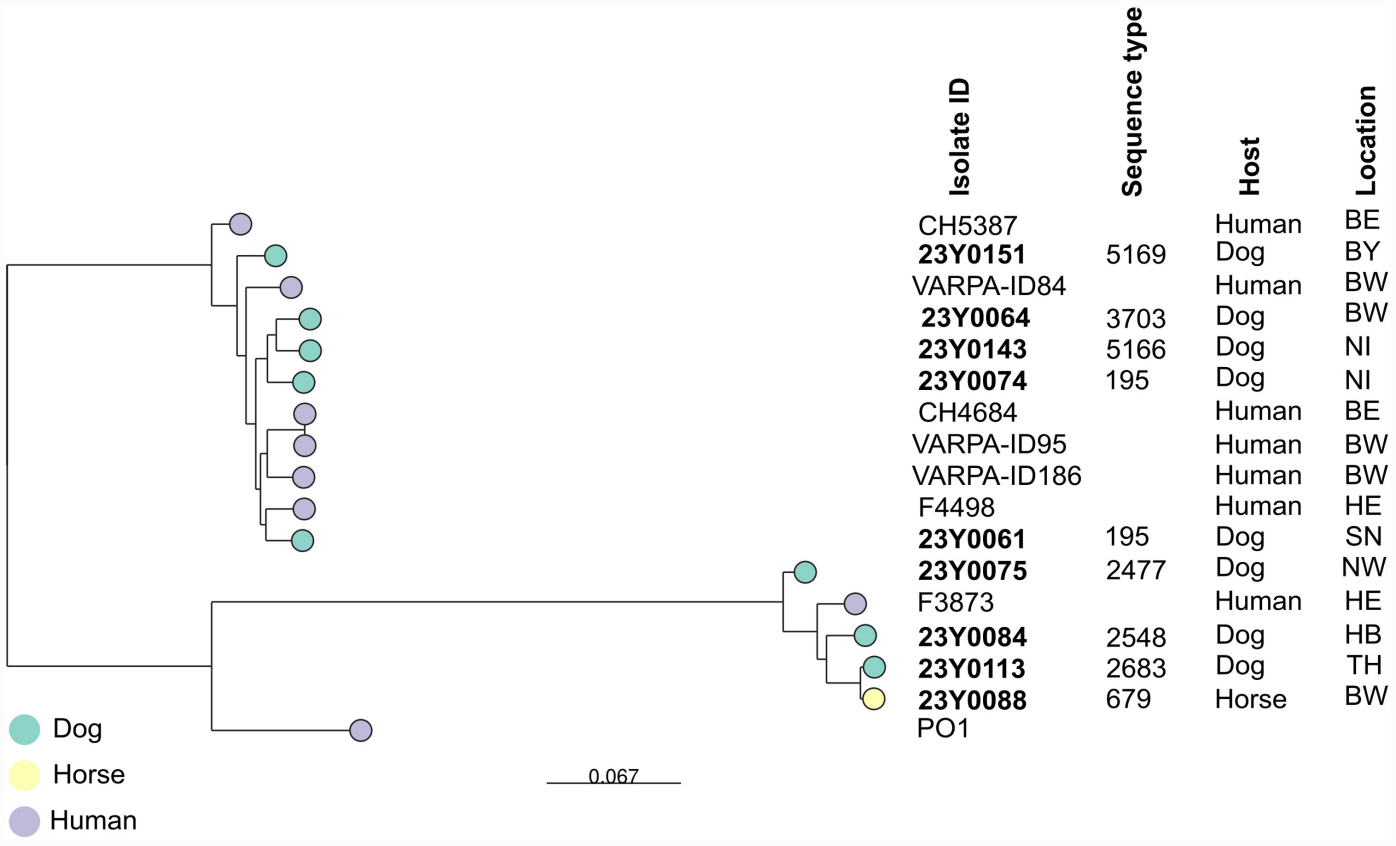
Maximum likelihood phylogenetic tree of *P. aeruginosa* isolates from Germany based on cgSNP. Leaf colors indicate the host. Isolates from this study are labeled in bold, and MLST sequence types (STs: 5169, 3,703, 5,166, 195, 2,477, 2,548, 2,683, and 679) are indicated. The bar at the bottom indicates base changes per site (BW: Baden-Wuerttemberg; BY: Bavaria; BE: Berlin; HB: Bremen; HE: Hesse; NI: Lower Saxony; NW: North Rhine-Westphalia; SN: Saxony; TH: Thuringia).

Core-genome single-nucleotide polymorphism (cgSNP) analysis showed considerable genetic diversity among the isolates. However, two isolates from dogs, one from Lower Saxony (23Y0081) and the other from North Rhine-Westphalia (23Y0008), were genetically identical (cgSNP difference = 0). Both belonged to ST4694 and carried the same number of AMR and virulence genes. Additionally, two further dog isolates from Bavaria (23Y0071) and North Rhine-Westphalia (23Y0077) showed close genetic relatedness (cgSNP difference = 7), were assigned to ST258, and displayed identical AMR profiles along with the same number of AMR and virulence genes. Due to the large number and variety of isolates, separate phylogenetic trees were created to provide a clearer visualisation of the genetic relationships (see Figures 3, 4). Several isolates clustered with *P. aeruginosa* isolates of human origin across Germany (Supplementary Table S1; Figures 3, 4). For example, a dog isolate from Brandenburg (23Y0066; HRC ST244) showed genetic relatedness to a human isolate from Hesse, differing by 15 cgSNPs. Likewise, a cat isolate from Bavaria (23Y0174; HRC ST395) showed genetic relatedness to a human isolate from Baden-Wuerttemberg, differing by 16 cgSNPs. Another cat isolate from North Rhine-Westphalia (23Y0171; HRC ST395) was genetically related to a human isolate from Baden-Wuerttemberg, differing by 21 SNPs.

## 4 Discussion

The current study provides a comprehensive genomic and phenotypic characterization of 72 *P. aeruginosa* isolates from various companion animal species across Germany. The isolates were obtained from various sampling sites, predominantly from dog ear infections. This finding is in agreement with previous studies that identified *P. aeruginosa* as a common cause of otitis in dogs (Nielsen et al., 2022b; Newstead et al., 2025). The isolates were collected from a diagnostic laboratory and, therefore, may not accurately reflect the prevalence, or MDR rate in the general companion animal population. AST analysis revealed a high prevalence of MDR isolates, with 97.2% resistant to at least one antibiotic in three various antibiotic classes. This aligns with prior findings by Nolff et al. (2016), reporting a high prevalence of MDR *P. aeruginosa* in isolates from open wounds in dogs and cats, in contrast to another study by Feßler et al. (2022), which reported no MDR among *P. aeruginosa* isolates from dogs and cats. Such discrepancies in MDR prevalence may be influenced by several factors, including prior antibiotic treatment, the spectrum and number of antibiotics tested, and geographic origin. In the current study, we used MICRONAUT-S MDR MRGN screening plates that contain a defined set of antibiotics for all MDRGN bacteria, including antipseudomonal therapy and other antibiotics. These agents were included primarily for resistance profiling and comparative purposes, not for clinical use as antipseudomonal therapy. We observed high resistance rates to cefotaxime, chloramphenicol, and trimethoprim/sulfamethoxazole. The widespread use of these broad-spectrum antibiotics in companion animals may contribute to the elevated resistance rates observed. In contrast, susceptibility to key antipseudomonal agents such as piperacillin/tazobactam and ceftazidime was observed, indicating that effective therapeutic options are still available. Nonetheless, ongoing antimicrobial stewardship is crucial to prevent further resistance development (Bassetti et al., 2022). Carbapenem resistance was detected in four dog isolates from geographically distinct federal states (Lower Saxony, Baden-Wuerttemberg, Saxony-Anhalt, and North Rhine-Westphalia), consistent with a previous study from Germany by Dalponte et al. (2025), which reported one imipenem-resistant isolate among 51 *P. aeruginosa* isolates from dogs. Carbapenems are a key antibiotic class in human medicine, and are designated by the European Medicines Agency (EMA) as Category A (“Avoid”) and not permitted for routine veterinary use in the European Union (EU) (EMA, 2020). The detection of carbapenem-resistant strains in companion animals is a significant public health concern, and suggests the possible circulation of transferable resistance genes between humans, animals, and the environment (Fernandes et al., 2018; Despotovic et al., 2023).

MLST analysis revealed substantial genetic diversity among isolates, with 59 STs identified, including five novel and six MDR HRCs (ST308, ST244, ST277, ST395, ST253, and ST274). The detection of HRCs in companion animals raises concerns about the potential transmission of *P. aeruginosa* from humans to these animals. This is supported by cgSNP analysis results, showing genetic relatedness between certain companion animal and human isolates, and consistent with the hypothesis of shared *P. aeruginosa* populations between humans and animals (Fernandes et al., 2018). HRCs of *P. aeruginosa* are disseminated in hospital settings worldwide (Oliver et al., 2015) and have also been detected in environmental sources, including drinking water networks (Horikian et al., 2024). In Germany, HRCs have previously been reported in both human clinical isolates and the environment (Table 5).

**Table 5:** Overview of the high-risk *P. aeruginosa* clones (ST308, ST277, ST244, ST395, ST253, and ST274) identified in the current study and reported in previous studies in Germany.

MLST	Source	Location	Number of isolates	Year	References
ST308	Hospital environment	Tuebingen	8	2009–2012	Willmann et al. (2015)
	Human	Frankfurt	3	2013–2017	Weber et al. (2022)
	Human	Tuebingen	1	2017–2019	Martak et al. (2022)
	Human and hospital environment	Germany (NS)	27	2018–2020	Rosenboom et al. (2023)
	Hospital environment	Southern Germany	1	2019	Rath et al. (2024)
	Human	Germany (NS)	1	2019–2021	Kocer et al. (2024)
	Cat	Baden-Wuerttemberg	1	2023	Current study
ST277	Human and environment	Germany (NS)	22	2018–2020	Rosenboom et al. (2023)
	Dog	Hesse	1	2023	Current study
ST244	Human	Frankfurt	25	2013–2017	Weber et al. (2022)
	Human	Tuebingen	2	2017–2019	Martak et al. (2022)
	Human and environment	Germany (NS)	37	2018–2020	Rosenboom et al. (2023)
	Dog	Brandenburg	1	2023	Current study
	Dog	Baden-Wuerttemberg	1		
	Dog	Hesse	1		
ST395	Human	Frankfurt	14	2013–2017	Weber et al. (2022)
	Human	Cologne	4	2015–2019	Wendel et al. (2022)
	Hospital environment	Tuebingen	2	2017–2019	Martak et al. (2022)
	Human and hospital environment	Germany (NS)	34	2018–2020	Rosenboom et al. (2023)
	Human	Germany (NS)	6	2019–2021	Kocer et al. (2024)
	Cat	North Rhine-Westphalia	1	2023	Current study
	Cat	Baden-Wuerttemberg	1		
	Cat	Bavaria	1		
ST253	Human	Frankfurt	2	2013–2017	Weber et al. (2022)
	Human and hospital environment	Tuebingen	6	2017–2019	Martak et al. (2022)
	Human and environment	Germany (NS)	39	2018–2020	Rosenboom et al. (2023)
	Human	Germany (NS)	4	2019–2021	Kocer et al. (2024)
	Dog	Lower Saxony	1	2023	Current study
	Dog	North Rhine-Westphalia	1		
	Dog	Schleswig-Holstein	1		
ST274	Human	Frankfurt	2	2013–2017	Weber et al. (2022)
	Human	Cologne	3	2015–2019	Wendel et al. (2022)
	Human and environment	Germany (NS)	36	2018–2020	Rosenboom et al. (2023)
	Human	Germany (NS)	2	2019–2021	Kocer et al. (2024)
	Dog	North Rhine-Westphalia	1	2023	Current study

MLST, multilocus sequence typing; ST, sequence type; NS, not specified.

The presence of AMR genes linked to multiple resistance mechanisms, including antibiotic inactivation, efflux pumps, target alteration, and reduced membrane permeability, underscores the diverse mechanisms contributing to AMR in German *P. aeruginosa* (Giovagnorio et al., 2023). Although all isolates harboured the *fos*A gene, conferring resistance to fosfomycin, only 26.4% were phenotypically resistant to fosfomycin. This finding suggests that the *fos*A gene alone may not be sufficient to confer fosfomycin resistance, and additional regulatory or expression factors might affect fosfomycin susceptibility (Laborda et al., 2022). Chromosomal *fos*A genes provide high-level resistance to fosfomycin in Gram-negative bacteria (Ito et al., 2017a). However, they do not confer resistance in 73.6% of current *P. aeruginosa* isolates. Some studies have shown that *fos*A inhibitors, like sodium phosphonoformate (PPF), can block the activity of chromosomally encoded *fos*A in certain Gram-negative strains (Ito et al., 2017b). Notably, some of these *fos*A inhibitors are already approved as antiviral drugs. Exposure to such inhibitors may explain why some strains remain susceptible to fosfomycin despite carrying the *fos*A gene (Wareth et al., 2022). Similarly, approximately 10% of isolates harboured a point mutation (V15I) in the *pmr*B gene, which mediates colistin resistance (Bolard et al., 2019); however, all isolates remained phenotypically susceptible at increased exposure to colistin.

The virulence profiling of isolates revealed a wide range of virulence factors. The most prevalent factors were associated with effector delivery systems that secrete proteins promoting immune evasion and supporting bacterial colonisation (Sharma et al., 2017). Among these, the Type III secretion system (T3SS) effector proteins, *exo*S, *exo*T, *exo*Y, and *exo*U were identified, contributing to the disruption of host cell signaling, cytotoxicity, and immune evasion (Sousa et al., 2021). Other important virulence determinants, including the pore-forming toxin *exl*A, which disrupts host cell membranes (Liao et al., 2022), and the *tox*A gene, which regulates exotoxin A synthesis and inhibits host protein synthesis (Qin et al., 2022), were also identified. The diversity of virulence factors in *P. aeruginosa* facilitates adaptation to various host environments, immune evasion, and successful colonisation (Liao et al., 2022). Plasmid analysis revealed that 27.8% of isolates carried plasmid contigs, of which 25% belonged to HRCs. Plasmids facilitate the horizontal transfer of AMR genes among bacterial populations (Odumosu et al., 2016). Furthermore, a class 1 integron carrying two integron-associated genes (*aad*A7 and *sul*1) was identified in a single HRC (ST253)- dog isolate, demonstrating that *P. aeruginosa* from dogs can harbour mobile genetic elements that contribute to the dissemination of antibiotic resistance. This finding aligns with a prior study reporting the same two integron-associated genes in dog isolates (Secker et al., 2025).

Although genome sizes varied among isolates, the number of AMR and virulence genes did not differ significantly. This variation in genome sizes may be attributed to the accessory genome (Klockgether et al., 2011), which accounted for 62% of the total genome in the current study, consistent with previous studies reporting that the accessory genome represents 75% of the total *P. aeruginosa* genome (Mosquera-Rendón et al., 2016). Generally, *P. aeruginosa* harbours a wide range of accessory genes, many of them are acquired by horizontal gene transfer mediated through mobile genetic elements (Freschi et al., 2019). These accessory genes often carry antibiotic resistance and virulence determinants, and such flexibility highlights the ability of *P. aeruginosa* to adapt to various environments and acquire traits that increase its pathogenic potential and antibiotic resistance (Dechathai et al., 2025).

## 5 Conclusion

The current study identified high genetic diversity among *P. aeruginosa* isolates from companion animals across Germany, including five novel STs (ST5165–ST5169) and six MDR high-risk clones. Several isolates showed genetic relatedness to *P. aeruginosa* strains recovered from humans in Germany; this finding highlights the potential transmission of *P. aeruginosa* between human and animal populations and emphasises the importance of the One Health approach in studying *P. aeruginosa* in Germany. Almost all isolates were MDR and carried a wide variety of AMR genes conferring resistance to *β*-lactams, aminoglycosides, fluoroquinolones, fosfomycin, and sulfonamides. However, a discrepancy between the phenotype and genotype was observed in some isolates.

Carbapenem-resistant strains were detected in four dog isolates from geographically separated federal states, despite carbapenems not being authorized for routine veterinary use in the EU. The presence of a diverse array of virulence factors and mobile genetic elements, such as class 1 integrons and plasmid contigs, in isolates of animal origin highlights the potential horizontal transmission of AMR genes. Overall, these findings underscore the need for continuous surveillance and stringent antimicrobial stewardship in veterinary medicine to mitigate the spread of AMR. Further research is needed to understand the drivers of resistance in animal-origin *P. aeruginosa*, develop effective prevention and control strategies, and reduce the risk of zoonotic transmission.

## Data Availability

The datasets presented in this study can be found in online repositories. The names of the repository/repositories and accession number(s) can be found in the article/Supplementary material.

## Footnotes

1
https://github.com/tseemann/snippy

2
https://github.com/egonozer/in_silico_pcr

## Supplementary material

Supplementary Table S1 The summary of WGS data, metadata, MLST, AST results, AMR genes, Virulence genes, plasmid analysis, and SNP typing of 72 *P. aeruginosa* isolates.
